# Optical imaging of metabolic dynamics in ALS under methionine regulation

**DOI:** 10.1117/1.JBO.30.S2.S23906

**Published:** 2025-05-24

**Authors:** Khang Hoang, Sirasit Prayotamornkul, Chan-yu Kuo, Hongje Jang, Lingyan Shi

**Affiliations:** a University of California San Diego, Shu Chien-Gene Lay Department of Bioengineering, La Jolla, California, United States; b University of California San Diego, Aiiso Yufeng Li Family Department of Chemical and Nano Engineering, La Jolla, California, United States; c University of California San Diego, Electrical and Computer Engineering, La Jolla, California, United States; d University of California San Diego, Institute of Engineering in Medicine, La Jolla, California, United States; e University of California San Diego, Synthetic Biology Institute, La Jolla, California, United States

**Keywords:** amyotrophic lateral sclerosis, TDP-43, L-methionine, stimulated Raman scattering microscopy, two-photon excitation microscopy, heavy water

## Abstract

**Significance:**

Excessive reactive oxygen species (ROS) in dysfunctional mitochondria, combined with inefficient antioxidant defenses, can drive amyotrophic lateral sclerosis (ALS) progression. L-methionine (Met) can neutralize ROS by modulating metabolism and activating antioxidants; however, its impact on ALS remains unknown.

**Aim:**

We aim to investigate the influence of excess Met on cellular metabolism and ROS accumulation and its role in ALS using multimodal optical imaging techniques.

**Approach:**

We applied deuterium oxide–probed stimulated Raman scattering imaging to study metabolic changes of lipids, proteins, and cytochrome c and two-photon excitation fluorescence imaging to assess mitochondrial redox state (nicotinamide adenine dinucleotide and flavin adenine dinucleotide ratio) in ALS cellular models under excess Met treatment. With three-dimensional (3D) image reconstruction, we investigated morphological changes of lipid droplets (LDs) and stress granules (SGs) in ALS models.

**Results:**

Excess Met not only promoted syntheses of lipids and unsaturated lipid membranes but also reduced protein synthesis, cytochrome c oxidation, and oxidative stress. Moreover, 3D image reconstruction showed that LDs increased in volume and number to promote cellular repair, whereas SGs decreased in volume but increased in number in response to reduced cellular stress.

**Conclusions:**

Excess Met offers a protective mechanism against oxidative stress and promotes cellular repair in ALS.

## 1 Introduction

Amyotrophic lateral sclerosis (ALS) is a fatal type of motor neuron disease characterized by a progressive loss of nerve cells in the brain and spinal cord. Following the diagnosis of ALS, only 5% to 10% of cases survive for another 15 to 18 years.^1^ Although the molecular mechanism of ALS is still unknown, elevated levels of reactive oxygen species (ROS) and inefficient antioxidant defense mechanisms have been associated with the progression of the disease.^2^^,^^3^ Excess cellular levels of ROS can harm proteins, nucleic acids, lipids, and membrane-bound organelles. Without an effective antioxidant defense mechanism, the continuous increase in ROS levels can trigger the release of apoptotic signals from the mitochondria, ultimately leading to neuronal death.^4^ The accumulation of ROS in ALS is caused by the translocation of transactive response DNA-binding protein 43 (TDP-43) from the nucleus into the mitochondria.^5^ Under normal conditions, TDP-43 shuttles between the nucleus and the cytoplasm, with a minor portion entering the mitochondria. However, in ALS patients, TDP-43 misfolds, truncates, and forms protein aggregates, resulting in its accumulation in the mitochondria which impairs mitochondrial function. One potential strategy to mitigate the onset of ALS is the use of antioxidant amino acids, such as L-methionine (Met), that can scavenge ROS.

**Fig. 1:**
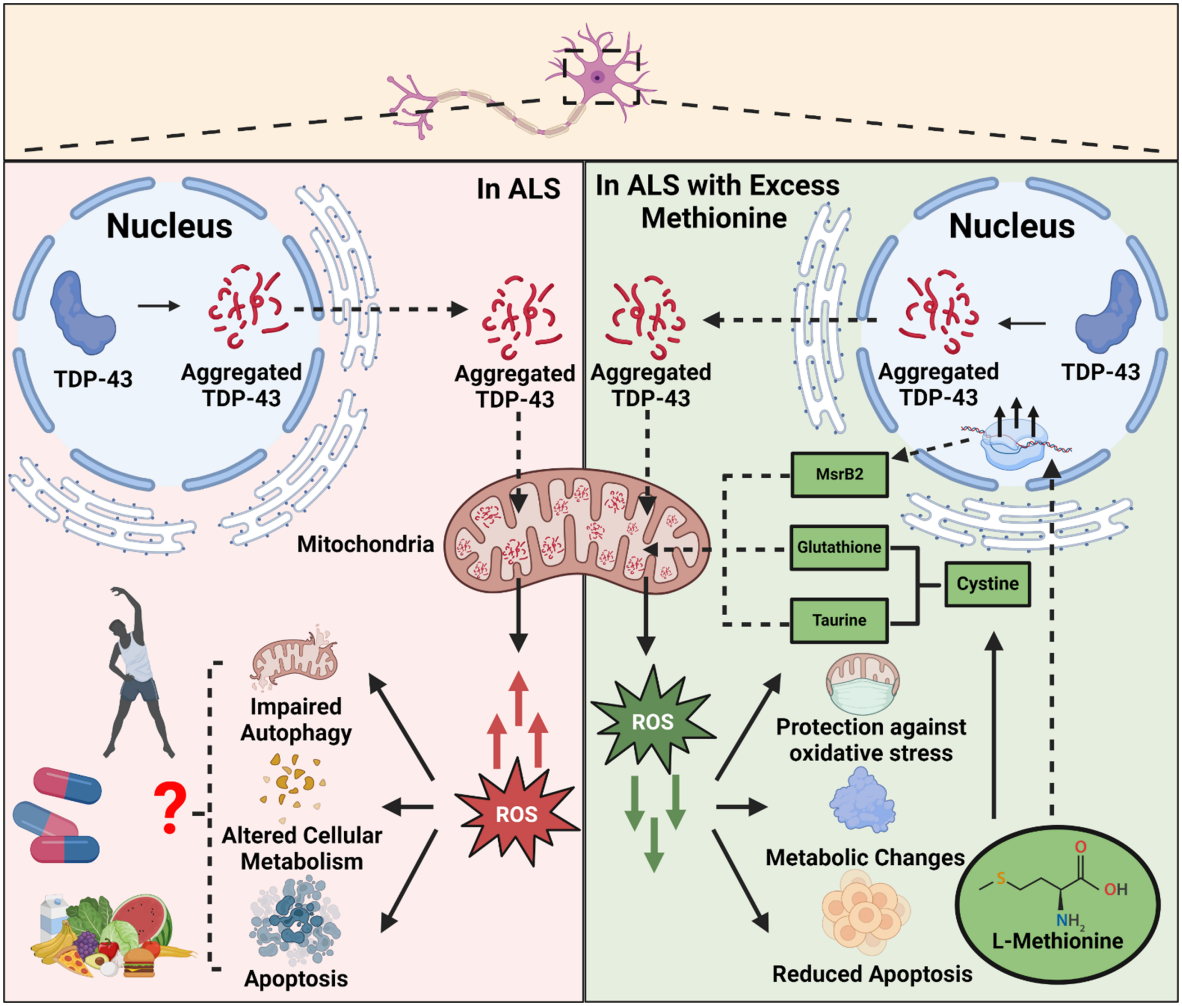
Impact of excess methionine levels on oxidative stress in amyotrophic lateral sclerosis.

Met ($C_{5}H_{11}{\mathrm{NO}}_{2}S$), an essential amino acid with a sulfur atom in its side chain, plays a crucial role in mammalian cell functions, such as cell proliferation and metabolism.^6^ As an antioxidant, Met can be oxidized to form methionine sulfoxide (MetO) through its sulfur atom. Subsequently, the enzyme methionine sulfoxide reductase can catalyze the reduction of MetO back to Met.^6^ In addition, Met can be converted to cysteine, which is a precursor for the synthesis of glutathione and taurine. Glutathione can be oxidized to glutathione disulfide (GSSG) by ROS. Then, the enzyme glutathione reductase can catalyze the reduction of GSSG back to glutathione.^7^ Through these oxidation–reduction cycles, both Met and glutathione help consume ROS, thereby reducing oxidative stress. Taurine is considered to control ROS through maintaining the normal activity of the electron transport chain, reducing inflammation, preventing the release of apoptotic signals from mitochondria, and sustaining glutathione synthesis.^8^ Furthermore, Met and its metabolite, S-adenosylmethionine (SAM), regulate protein and lipid synthesis through the mTOR pathway, which in turn enhances the translation of mRNA into proteins and upregulates lipogenic enzymes essential for fatty acid and triglyceride synthesis.^9^ These processes also play a crucial role in helping cells recover from oxidative stress. However, it remains unclear whether excess Met regulation can effectively reduce oxidative stress and rescue altered metabolic changes in ALS (Fig. 1).

To investigate cellular metabolic activities, mass spectroscopy (MS)-based imaging techniques such as electrospray ionization–MS and liquid chromatography–MS have been implemented. However, these methods are destructive to live biological samples and result in the loss of *in situ* spatial information of biomolecules.^9^ Raman spectromicroscopy has been employed for cellular and molecular chemical imaging due to its non-invasive nature, high subcellular resolution, and excellent chemical specificity. We developed a novel, multimodal imaging platform, combining deuterium oxide–probed stimulated Raman scattering (DO-SRS) microscopy and two-photon excitation fluorescence (2PEF) microscopy to investigate the metabolism of a variety of biomolecules in cells and living organisms.^10^^–^^16^ Deuterium (D) ions of heavy water can be harnessed and integrated into newly synthesized macromolecules via a series of enzymatic reactions, forming carbon–deuterium (C-D) bonds that are detectable by Raman spectroscopy.

In this study, we utilized the DO-SRS and 2PEF multimodal imaging platform to characterize the antioxidant effects of a methionine-enriched diet on TDP-43-expressed human embryonic kidney (HEK293) cells. DO-SRS imaging was applied to quantify lipid and protein metabolism, whereas 2PEF autofluorescence imaging was used to examine changes in flavin adenine dinucleotide (FAD) and nicotinamide adenine dinucleotide (NADH). The optical redox ratio (NADH/FAD) reflects the ROS levels in biological samples and provides insights into the metabolic activity, specifically the oxidation-reduction states, of the cells. Furthermore, label-free images of unsaturated lipid, saturated lipid, and cytochrome c (Cyt c) by stimulated Raman scattering (SRS) microscopy reveal mitochondrial metabolism. In addition, 3D SRS images of subcellular organelles, including lipid droplets (LDs) and stress granules (SGs), further elucidate the progression of ALS under excess Met treatment.

## 2 Materials and Methods

### 2.1 Cell Culture

Human embryonic kidney 293 (HEK293) cells of passage 1 were obtained from Dr. Jane Wu’s Lab at Northwestern University. HEK293 was used as a control. T-Rex-293 cells (Invitrogen, Waltham, Massachusetts, United States) derived from HEK293 were transfected with pcDNA4 TO/myc-His plasmids (Invitrogen) expressing either wild-type (Wt) or an ALS-associated TDP-43 mutant (A315T). The induction of TDP-43 expression was thoroughly validated in a previous study.^4^ By adopting the same culture and induction protocol from this study, we can reasonably infer that the expression profile remains unchanged.

All three cell lines were cultured in Dulbecco’s modified Eagle medium (DMEM, Corning, Prod. No. 10-013-CV, Corning, New York, United States) supplemented with 10% fetal bovine serum (FBS, Gibco, Cat. No. A3840002, Gibco, Billings, Montana, United States) and 1% penicillin-streptomycin (P/S, Gibco, Cat. No. 15140122). At 80% confluence and greater than 90% viability after at least two passages since thawing, cells were washed with 1× phosphate-buffered saline without magnesium and calcium ions (PBS, Gibco, Cat. No. 14190144) and then detached using 0.05% Trypsin-EDTA (Gibco, Cat. No. 25300120). Following detachment, cells were centrifuged, and the supernatant was removed to obtain a cell pellet. This pellet was then resuspended in fresh DMEM and seeded at a concentration of $2\times 10^{5}\text{ cells}/\mathrm{mL}$ onto sterile laminin-coated coverslips in a 24-well plate. After 12 h, the cells were synchronized for 6 h by changing the culture medium to DMEM supplemented with 0.5% FBS and 1% P/S. Then, Wt and A315T-mutant TDP-43 cells were incubated in DMEM containing 0.25  μg/mL tetracycline (Tet), 5% FBS, and 1% P/S for 36 h to induce TDP-43 expression. Simultaneously, the control HEK293 cells were cultured in DMEM supplemented with 5% FBS and 1% P/S but without Tet. After that, all cells were treated in DMEM culture media containing 5% FBS, 1% P/S, and 50% (v/v) $D_{2}O$ (Cambridge Isotope Laboratories, Cat. No. 7789-20-0, Tewksbury, Massachusetts, United States) supplemented with either excess (2×, 60  mg/L) or regular (1×, 30  mg/L) methionine (Met, Sigma Aldrich, Cat. No. M9625, St. Louis, Missouri, United States) and incubated for 48 h. The Met concentrations were selected based on a previous study in which 100  mg/L Met displayed 95% cell viability and did not artificially elevate ROS levels.^17^ Then, cells were fixed with 4% paraformaldehyde (PFA, VWR, Cat. No. 15713-S) for 10 min or 99.9% methanol (MeOH, Millipore Sigma, Cat. No. 646377, Burlington, Massachusetts, United States) for 18 min at room temperature. The coverslips were finally mounted on 1-mm-thick glass microscope slides with 9 mm diameter and 120-μm-thick spacers filled with 1× PBS with magnesium and calcium ions (Gibco, Cat. No. 14040141) for imaging and spectroscopy measurements. The samples were stored at 4°C when not in use. A detailed cell culture diagram is shown in Fig. S1 in the Supplementary Material.

### 2.2 Spontaneous Raman Spectroscopy

**Fig. 2:**
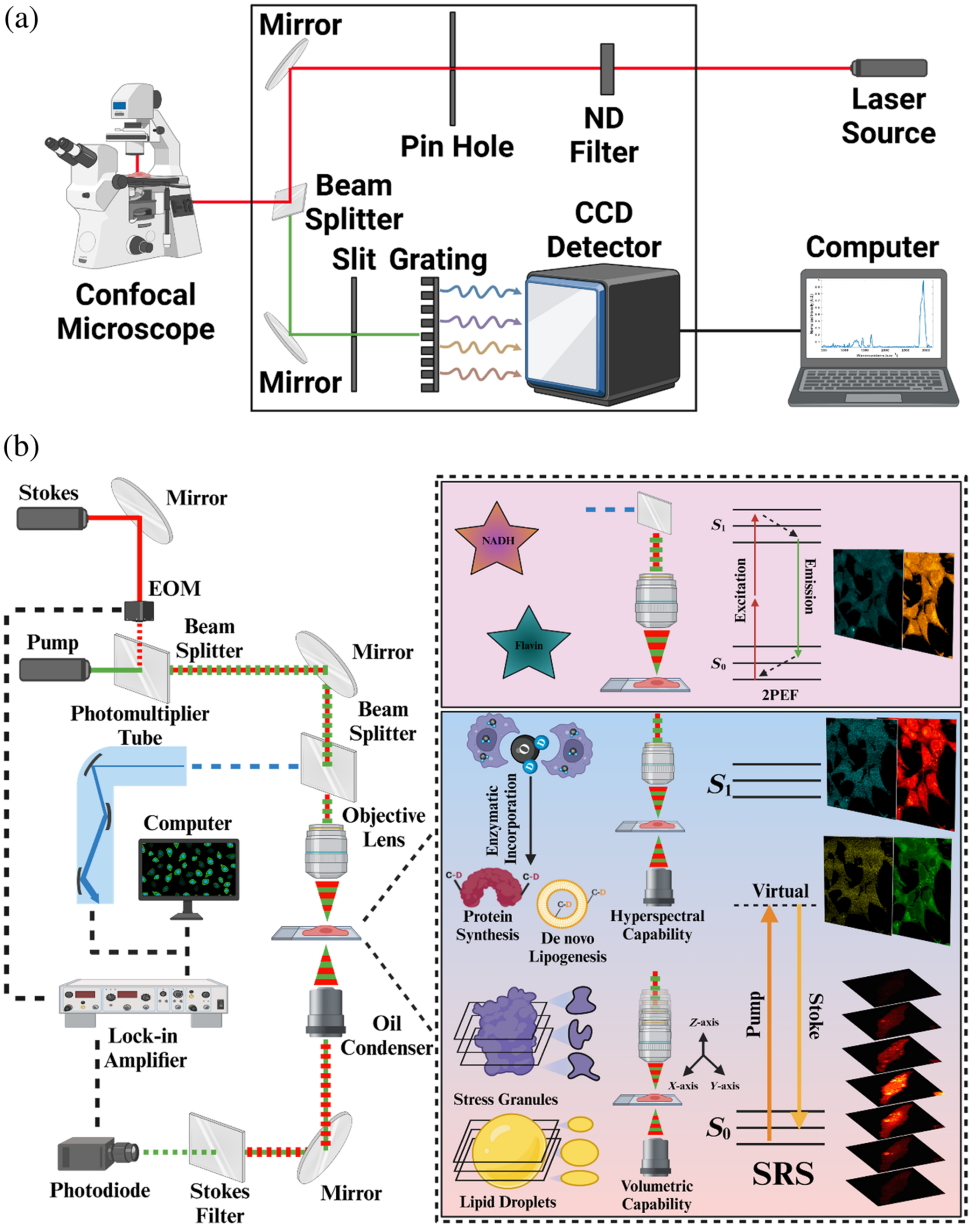
Schematic of spontaneous Raman spectroscopy, DO-SRS microscopy, and 2PEF microscopy. (a) Spontaneous Raman spectroscopy configuration. (b) DO-SRS, 2PEF, and volumetric SRS configuration.

A confocal Raman microscope (XploRA PLUS, Horiba, Kyoto, Japan) was used to obtain spontaneous Raman spectra. The microscope is equipped with a 532-nm diode laser source with 1800  lines/mm grating. The excitation power is 40 mW after passing through a 100× objective lens (MPLN 100×, Olympus, Tokyo, Japan). The spectrometer collects the intensity values in each region of interest from 400 to $3150\;{\mathrm{cm}}^{-1}$. The acquisition time used for these samples was 90 s with a binning of 4 and an accumulation of 3. Each spectrum was acquired by targeting desired subcellular regions including lipid droplets and nuclei, and an additional spectrum was taken of the background with PBS in the same focal plane through the LabSpec6 acquisition software (Horiba). Afterward, the background was subtracted from the subcellular target spectrum to obtain background-free data. The background-free data were subjected to data analysis. A detailed configuration of spontaneous Raman spectroscopy is shown in Fig. 2(a).

### 2.3 SRS Microscopy

An upright laser-scanning microscopy (DIY multiphoton, Olympus) with a 25× water object (XLPLN, WMP2, 1.05 NA, Olympus) was used for near-IR throughput. The Stokes beam (applied physics and electronics) with a wavelength of 1031 nm, 6-ps pulse width, and 80-MHz repetition rate was coupled with a synchronized pulsed pump beam (applied physics and electronics) with a tunable 720- to 990-nm wavelength, 5- to 6-ps pulse width, and 80-MHz repetition rate into the microscopy. An oil condenser with 1.4 NA was used to collect Stokes and pump beams onto the sample. For the water-immersion objective lens, a large water droplet was placed on the sealed sample slide. The Stokes beam was blocked by a high O.D. short-pass filter (950 nm, Thorlabs, Newton, New Jersey, United States) and transmitted the pump beam onto an Si photodiode to detect the stimulated Raman loss signal. A lock-in amplifier at 20 MHz was utilized to terminate, filter, and demodulate the output current from the photodiode where the demodulated signal forms the image during the laser scanning. The FV3000 software module FV-OSR (Olympus) was used to display Raman images.

Images were collected at a resolution of 512×512  pixels using a dwell time of 100  μs/pixel, a time constant (TC) of 80  μs, a digital zoom of 5, and a 450-mW laser power. The images were saved as .OIR graphic files by the Olympus FluoView acquisition software. The background image was acquired at $1900\;{\mathrm{cm}}^{-1}$ and subtracted from all the SRS images using the ImageJ software. For multichannel SRS imaging, the pump wavelength was tuned so that the energy difference between the pump and Stokes beams matched the vibrational mode of a specific chemical bond. For volumetric SRS imaging, a discrete stack of z-depths encompassing the entire cell was identified. The microscope was then configured to scan through the z-stack in a 1-μm increment to create a 3D model of the cell. The images were collected at a resolution of 512×512  pixels with a dwell time of 80  μs/pixel, a TC of 60  μs, a digital zoom of 10, and a 300-mW laser power. A detailed configuration of multimodal DO-SRS and 2PEF is shown in Fig. 2(b).

### 2.4 Two-Photon Excitation Fluorescence Microscopy

Label-free autofluorescence of NADH and FAD was excited at 780 and 860 nm, respectively, using the same tunable picosecond laser. Backscattered emission of these molecules was collected using a 460-/515-nm filter cube (OCT-ET460/50M32, Olympus). These images were acquired at 512×512  pixels, 12.5-μs/pixel using a 450-mW laser power. The 2PEF microscopy configuration is shown in Fig. 2(b).

### 2.5 Data Analysis

#### 2.5.1 Spectral analysis

The spectra were processed using LabSpec6, MATLAB, and OriginPro. Raman spectra of subcellular regions were background-subtracted with Raman spectra of PBS background on LabSpec6. The resulting spectra were interpolated at every wavenumber (${\mathrm{cm}}^{-1}$) and converted into an array. The raw spectrum data were graphed for verification in the MATLAB software (version 2024a). Then, baseline correction and vector normalization with the intensity of the ${\mathrm{CH}}_{3}$ Raman band at $2930\;{\mathrm{cm}}^{-1}$ were performed. The processed spectra were averaged into a single spectrum to reduce noise. Each spectral peak was later labeled and assigned to the vibrations of a particular chemical bond on Origin 2024b (version 10.15).

#### 2.5.2 Image analysis

Two-dimensional (2D) Raman hyperspectral images were processed using MATLAB and ImageJ. The images were background-subtracted with their corresponding background images acquired at $1900\;{\mathrm{cm}}^{-1}$. Sixty individual cells in Raman hyperspectral and autofluorescence images from three separate trials were manually segmented and measured for their average absolute intensity. These cells were relative in sizes and shapes and randomly selected for analysis.

3D image stacks of lipid droplets and stress granules taken at 2850 and $2930\;{\mathrm{cm}}^{-1}$, respectively, were processed using MATLAB and ImageJ. Volumetric pixel (voxel) dimensions, including width, height, and depth in micrometers, were extracted from the 3D stacks. The intensity values in each z-plane were normalized using the global minimum and maximum intensities across all slices to ensure consistent intensity ranges. Detected lipid droplets and stress granules were identified using a circular Hough transform–based algorithm, which could locate circular regions within a specified size range.^18^^,^^19^ These regions were converted into binary masks and connected across the z-stack using 3D connected component labeling to account for individual droplets that span multiple slices. The volume of each droplet was then calculated using voxel-based measurements, where the total volume was determined by multiplying the number of voxels in a connected region by the physical volume of a single voxel

$$\text{Volume of lipid droplets and stress granules}=V_{\text{Single voxel}}\times \text{no. of voxels}.$$ (1)

This approach counts the 3D pixels of each detected droplet to ensure accurate measurement of those with irregular shapes. After processing the 3D image stacks with MATLAB to detect and analyze stress granules, ImageJ was used to verify the results. This included measuring the radii and volumes of the identified organelles and ensuring that the granules detected by MATLAB corresponded to their true locations and sizes as observed in the original 3D images. Given the large number of lipid droplets detected by MATLAB and observed by ImageJ, manually verifying the results was impractical. However, the detected stress granules closely matched their true locations, leading to the assumption that the lipid droplet calculations were also accurate.

#### 2.5.3 Statistical analysis

Three separate trials were conducted and included in the analysis. SRS and 2PEF images used in 2D multimodal analysis consist of four ROIs of ∼10 cells per ROI in each experimental group per trial. Five cells from each ROI were then manually segmented and measured for their average absolute intensity. SRS images used in 3D image reconstruction of subcellular organelles consist of two ROIs of a single cell per experimental group per trial. Subcellular organelles were analyzed by MATLAB and ImageJ as described above. The results of three separate trials were pooled into one large dataset rather than using the mean of individual trials.

Statistical significance among experimental groups in the dataset was compared using two-way ANOVA (GraphPad Prism, version 10.2.3). The data that had p-values lower than 0.05 (p<0.05) were identified as statistically significant.

## 3 Results

### 3.1 DO-SRS Imaging of Protein and Lipid Syntheses

To investigate whether excess methionine treatment would enhance cellular repair and rescue cells expressing TDP-43 from ALS, we examined lipid and protein metabolism in cells using DO-SRS imaging.

**Fig. 3:**
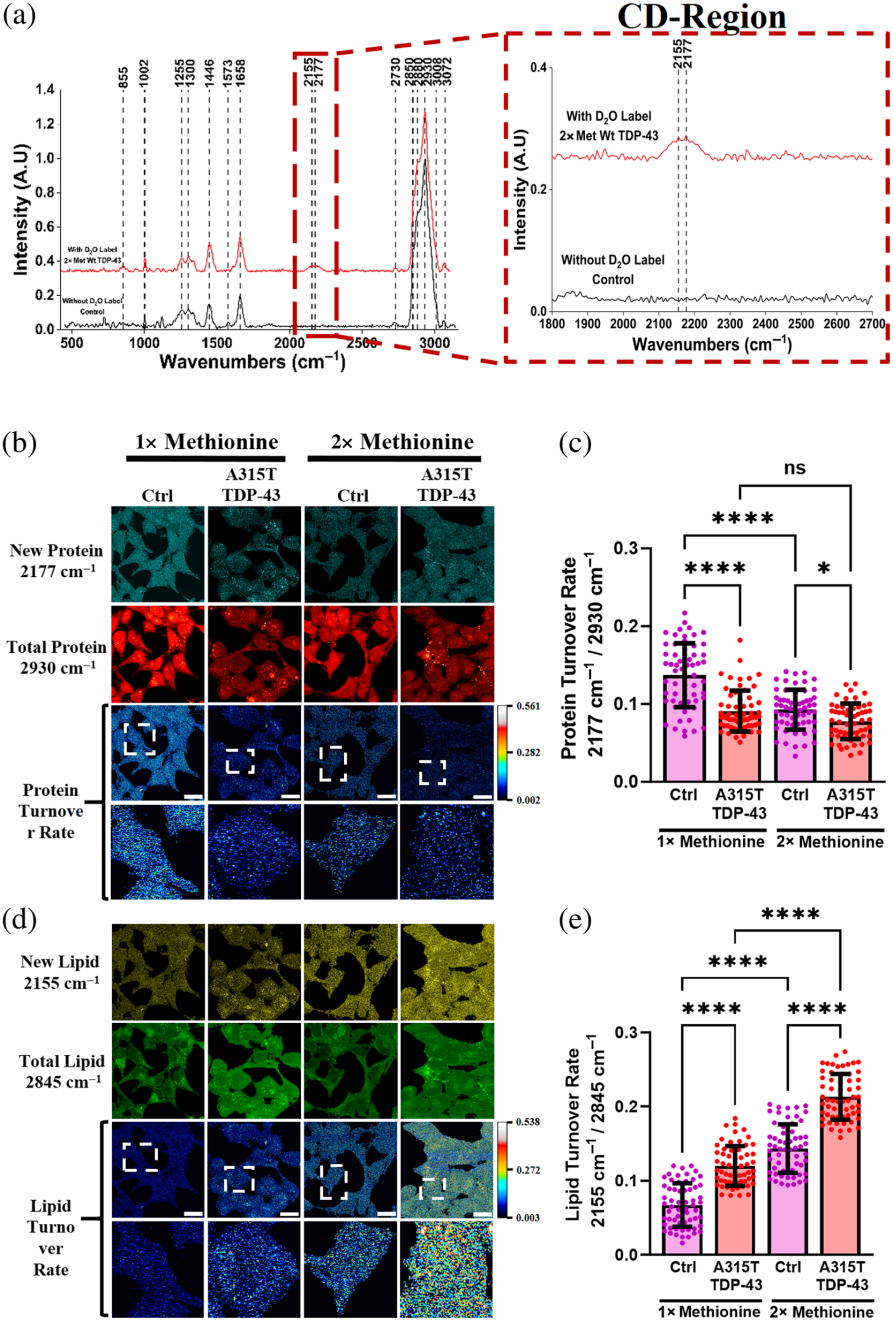
DO-SRS imaging of deuterated lipids and proteins in control (Ctrl) and an ALS-associated A315T cell samples under methionine regulation. (a) C-D signals in newly synthesized macromolecules emerged in the Ctrl and A315T-mutant TDP-43 cells incubated in 50% $D_{2}O$ medium for 48 h. The spontaneous Raman spectrum displayed in red revealed the C-D signals of newly synthesized lipids and proteins at 2155 and $2177\;{\mathrm{cm}}^{-1}$, respectively. In contrast, the spontaneous Raman spectrum of control cells grown in a regular medium without 50% $D_{2}O$ in black did not show the emergence of C-D signals. (b) and (d) DO-SRS images illustrate cells cultured in regular (1×, 30  mg/L) or excess (2×, 60  mg/L) methionine medium, both supplemented with 50% $D_{2}O$ for 48 h. Newly synthesized lipids were visualized at $2155\;{\mathrm{cm}}^{-1}$, whereas newly synthesized proteins were visualized at $2177\;{\mathrm{cm}}^{-1}$. Dashed outlines highlight single cells, revealing metabolic heterogeneity in the synthesis of new proteins and lipids. Specifically, newly synthesized proteins are either concentrated in the cytoplasm or nucleus, whereas newly synthesized lipids are distributed throughout the entire cell. (c) and (e) Quantitative analysis demonstrates the turnover rates of proteins (c) and lipids (e) calculated by dividing the amount of newly synthesized macromolecules (either at 2155 or $2177\;{\mathrm{cm}}^{-1}$) by the total amount of existing and newly synthesized macromolecules (either at 2845 and $2930\;{\mathrm{cm}}^{-1}$) in the cells. Data are presented as mean ± SD, with N=60 cells per group. *p<0.05; **p<0.01; ***p<0.001; ****p<0.0001; ns, non-significant difference. Scale bar is 20  μm.

First, we grew cells in DMEM culture media supplemented with 50% $D_{2}O$ (v/v) and used spontaneous Raman spectroscopy to obtain spectra of LDs in the cells. LDs serve as essential hubs for cellular metabolism such as cellular repair and energy storage.^19^ With $D_{2}O$ treatment, the deuterium (D) ions would be incorporated into newly synthesized biomolecules through a series of enzymatic reactions, replacing carbon–hydrogen (C-H) bonds by C-D bonds. The C-D bonds display characteristic peaks in the cell silent region that can be detected by Raman spectroscopy. Here, we observed distinct Raman signals at 2155 and $2177\;{\mathrm{cm}}^{-1}$, correspondingly, the C-D bonds in newly synthesized lipids and protein, respectively [Figs. 3(a) and S2 in the Supplementary Material]. In contrast, these C-D signals were not observed in cells grown in regular media [Fig. 3(a)]. Moreover, another two distinct peaks were also identified at 2850 and $2930\;{\mathrm{cm}}^{-1}$, respectively, corresponding to the stretching vibration of C-H bonds in total lipid and protein (Fig. S2 in the Supplementary Material).

After identifying characteristic wavenumbers of C-D and C-H bonds in lipids and proteins, we captured SRS images of newly synthesized and total lipids/proteins inside single cells at these frequencies, respectively. We also generated ratiometric images and quantified the turnover rates of lipids and proteins by calculating the ratios of newly synthesized to total components: $2155/2850\;{\mathrm{cm}}^{-1}$ for lipids and $2177/2930\;{\mathrm{cm}}^{-1}$ for proteins. Increased cytoplasmic localization of TDP-43 has been reported to reduce global protein synthesis through the disruption of polyribosome activities and altered lipid metabolism.^20^ Our SRS images demonstrated significantly lower protein turnover rates in A315T-mutant TDP-43 cells, but not in Wt cells, under both regular (1×) and excess (2×) Met treatments when compared with control cells. In addition, excess Met treatment dramatically reduced protein turnover in both Wt and control cells but not in A315T-mutant TDP-43 cells [Figs. 3(b) and 3(c) and Figs. S3(a) and S3(b) in the Supplementary Material]. In contrast, lipid turnover rates in A315T-mutant TDP-43 and Wt cells were significantly higher than control cells under both regular and excess Met treatments, whereas excess Met elevated lipid turnover in all cells [Figs. 3(d) and 3(e) and Figs. S3(c) and S3(d) in the Supplementary Material]. These findings indicate that methionine influenced both protein and lipid turnover rates. However, the changes in lipid turnover rate were more evident than the protein turnover rate in all cells.

### 3.2 2PEF and SRS Imaging to Investigate Oxidative Stress, Lipid Unsaturation, and Cytochrome *c* Oxidation

**Fig. 4:**
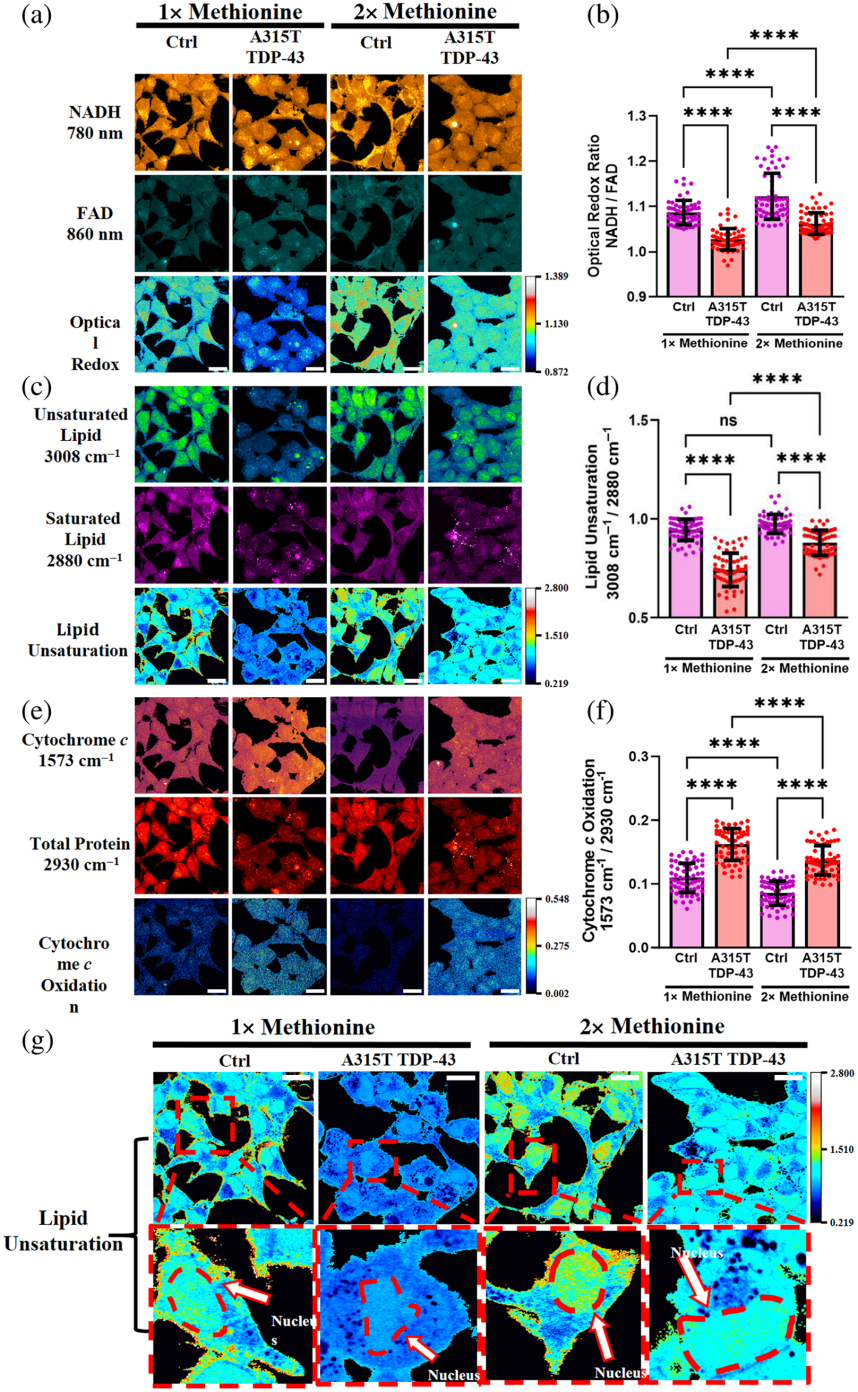
2PEF and SRS imaging of optical redox state, lipid unsaturation and cytochrome c in Ctrl, and ALS-associated A315T cell samples under methionine regulation. (a), (c), and (e) Label-free multichannel 2PEF and SRS images to investigate oxidative stress in the Ctrl and A315T-mutant TDP-43 cells under regular (1×, 30  mg/L) methionine or excess (2×, 60  mg/L) methionine medium. (b), (d), and (f) Quantitative analysis demonstrates optical redox ratio (b), lipid unsaturation (d), and cytochrome c oxidation (f) in the cells. The optical redox ratio was determined by dividing the autofluorescence signal of NADH at 780 nm by the autofluorescence signal of FAD at 860 nm and used as an estimate of the ROS levels in the cells. To explore lipid peroxidation in cells under methionine regulation, the lipid unsaturation ratio was calculated by dividing the amount of unsaturated lipid detected at $3011\;{\mathrm{cm}}^{-1}$ by the amount of saturated lipid detected at $2880\;{\mathrm{cm}}^{-1}$. In addition, cellular apoptosis in cells under methionine regulation was assessed by dividing the amount of oxidized cytochrome c detected at $1573\;{\mathrm{cm}}^{-1}$ by the total protein content detected at $2930\;{\mathrm{cm}}^{-1}$ in the cells. Data are presented as mean ± SD, with N=60 cells per group for optical redox, lipid saturation, and cytochrome c under different methionine conditions. *p<0.05; **p<0.01; ***p<0.001; ****p<0.0001; ns, non-significant difference. Scale bar is 20  μm. (g) Dashed outlines highlight single cells that exhibit unique distribution of lipid unsaturation under methionine regulation. Specifically, excess methionine treatment upregulates lipid unsaturation around the nuclear region of the cells as shown by the arrows.

Using 2PEF microscopy, we captured autofluorescence images of NADH and FAD at 780 and 860 nm, respectively [Fig. 4(a) and Fig. S4a in the Supplementary Material]. The optical redox ratio, here defined as the intensity ratio of NADH to FAD, is commonly used to quantify the redox state in the cell.^21^^,^^22^ More specifically, increased ROS levels are associated with decreased NADH concentrations.^23^ This increase of NADH relative to FAD can serve as an indicator of reduced oxidative stress. From our measurements, we observed that there were significantly lower optical redox ratios in both A315T-mutant TDP-43 and Wt cells compared with control cells under both regular and excess Met treatments [Fig. 4(b) and Fig. S4(b) in the Supplementary Material]. In addition, excess Met significantly enhanced the optical redox ratios in all cells, implying reduced ROS levels.

We next investigated lipid unsaturation in cells by computing the ratio of unsaturated to saturated lipids. Unsaturated and saturated lipids were identified at 3008 and $2880\;{\mathrm{cm}}^{-1}$, respectively, in the Raman spectra (Fig. S2 in the Supplementary Material), and SRS images were captured at these frequencies [Fig. 4(c) and Fig. S4(c) in the Supplementary Material]. We measured declined lipid unsaturation levels in A315T-mutant TDP-43 and Wt cells under both regular (1×) and excess (2×) Met treatments compared with the control. Excess Met dramatically enhanced lipid unsaturation in A315T-mutant TDP-43 and Wt cells, but there were no evident differences among control cells subjected to regular and excess Met treatments [Fig. 4(d) and Fig. S4(d) in the Supplementary Material]. Upon excess Met treatment, Met and its metabolic conversion to glutathione and taurine are able to neutralize ROS,^6^^–^^8^ promoting the production of unsaturated fatty acids in the cells and supporting the conclusions derived from our optical redox ratio measurements. We also observed highly concentrated lipid unsaturation levels near the nucleus of single cells under excess Met treatment [Fig. 4(g)].

We further investigated mitochondrial dysfunction caused by ROS in TDP-43 expressing cells by assessing the metabolism of cytochrome c (Cyt c), a mitochondrial electron-transporting protein. Mitochondrial dysfunction, causing the release of oxidized Cyt c into the cytoplasm and inducing apoptosis, has been associated with ALS.^24^^,^^25^ Our Raman spectra revealed a strong peak at $1573\;{\mathrm{cm}}^{-1}$ in all three cell lines (Fig. S2 in the Supplementary Material), which is attributed to the heme group of oxidized Cyt c.^26^^,^^27^ This was verified by the remaining of this peak after lipids were removed with methanol wash [Fig. S4(g) in the Supplementary Material]. At this frequency, we captured SRS images of oxidized Cyt c within single cells [Fig. 4(e) and Fig. S4(e) in the Supplementary Material]. SRS images and quantification results showed that compared with control cells, Cyt c level was significantly elevated in A315T-mutant TDP-43 cells under regular Met treatment but not in Wt cells. Excess Met dramatically reduced Cyt c levels in both A315T-mutant TDP-43 and control cells, but not in Wt cells [Fig. 4(f) and Fig. S4(f) in the Supplementary Material]. This evidence suggests that the excess Met treatment reduces the extent of Cyt c oxidation by ROS in TDP-43-expressing cells.

### 3.3 3D SRS Imaging of Metabolism in Subcellular Organelles in Cells Expressing TDP-43

**Fig. 5:**
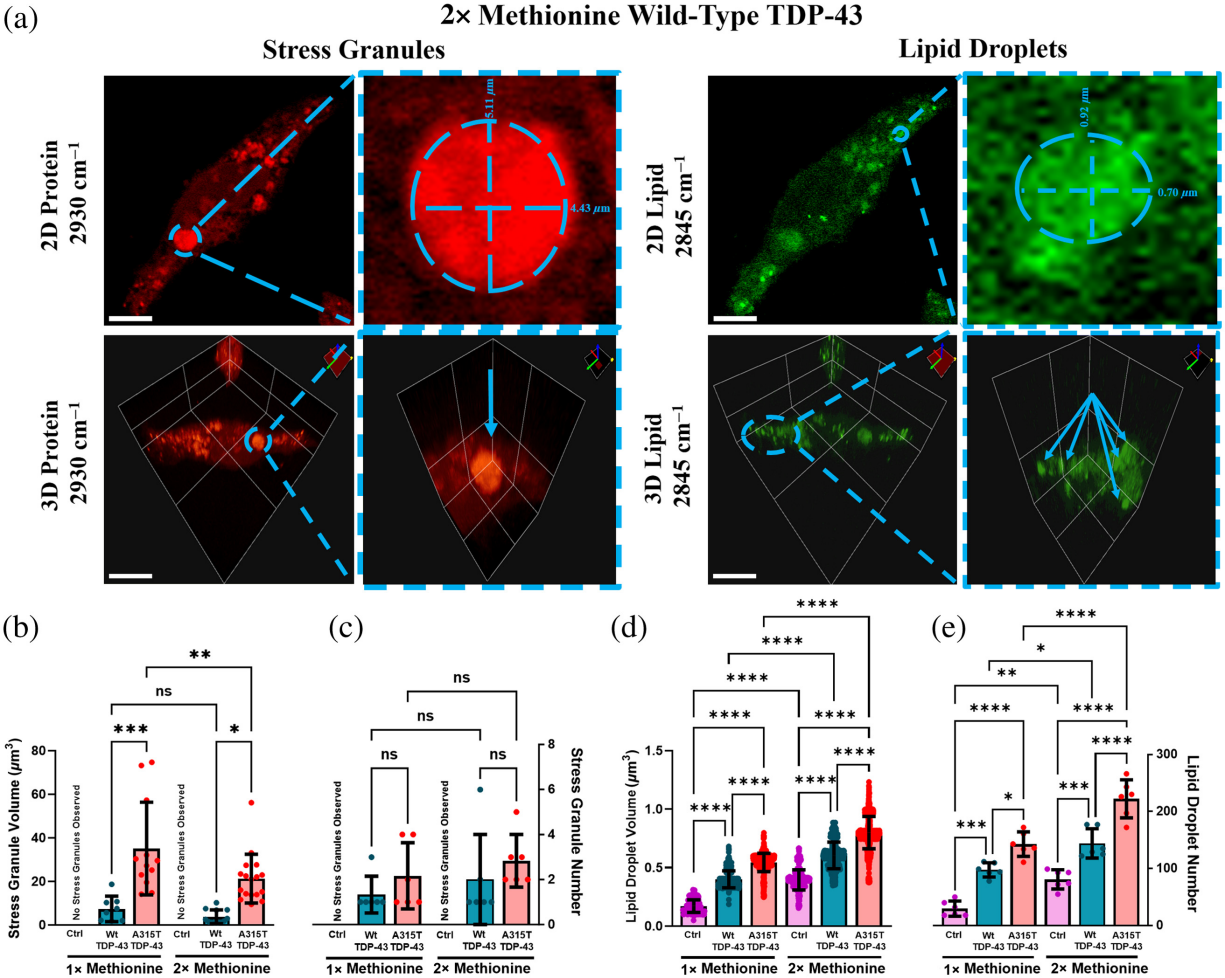
Analysis of subcellular organelles—stress granules and lipid droplets in Ctrl, wild-type (Wt) TDP-43, and ALS-associated A315T cell samples under methionine regulation. (a) 2D and 3D visualization of stress granules and lipid droplets within single cells by label-free SRS under regular (1×, 30  mg/L) methionine or excess (2×, 60  mg/L) methionine medium. The $2930\text{-}{\mathrm{cm}}^{-1}$ wavelength was used to observe protein-rich stress granules, whereas the $2850\text{-}{\mathrm{cm}}^{-1}$ wavelength was used to visualize lipid-rich lipid droplets. Dashed outline highlights a single stress granule or cluster of lipid droplets on the same single cell. (b) and (c) Quantitative analysis of the volume and number of stress granules in the cells. (d) and (e) Quantitative analysis of the volume and number of lipid droplets in the cells. Data are presented as mean ± SD, with N=6 individual cells per group for stress granules and lipid droplets under different methionine conditions. *p<0.05; **p<0.01; ***p<0.001; ****p<0.0001; ns, non-significant difference. Scale bar is 10  μm.

LDs are organelles that modulate lipid synthesis essential for cellular growth and proliferation.^19^^,^^28^ On the other hand, SGs store, degrade, and re-initiate protein translation of misfolded proteins.^29^^,^^30^ Although the expression of cytoplasmic TDP-43 has been found to accumulate LDs in size and number, little is still known about SG metabolism.^31^ By tuning the pump laser to $2850\;{\mathrm{cm}}^{-1}$ (for imaging lipids) and $2930\;{\mathrm{cm}}^{-1}$ (for imaging protein), we acquired 2D images of LDs and SGs within single cells [Fig. 5(a)]. We further achieved 3D images of LDs and SGs within the same cells by reconstructing stacks of 2D images along the z-direction with a step size of 1  μm [Fig. 5(a)] and then quantified the volume and number of LDs and SGs.

SRS images at $2930\;{\mathrm{cm}}^{-1}$ detected SGs as bright, rounded blobs with radii ranging from 2 to 6  μm [Fig. 5(a) and Fig. S5 in the Supplementary Material]. However, the SG signal significantly diminished in images taken at $2850\;{\mathrm{cm}}^{-1}$, indicating high protein contents in SGs. We further verified this by treating cells with methanol to wash out lipids and still observed the same SG signals (Fig. S6 in the Supplementary Material). Notably, we could not detect any SGs in the control cells without TDP-43 expression, suggesting that SGs were specifically induced by the expression of TDP-43 (Fig. S5 in the Supplementary Material).

SRS images captured at $2850\;{\mathrm{cm}}^{-1}$ showed LD clusters with radii ranging from 0.10 to 1.2  μm [Fig. 5(a) and Fig. S5 in the Supplementary Material]. Methanol wash effectively removed clusters of LDs on single cells, further confirming that these bright droplets were indeed high lipid content organelles (Fig. S5 in the Supplementary Material).

Under regular Met conditions, the volume of SGs in A315T-mutant TDP-43 cells was significantly larger compared with Wt TDP-43 cells. Similarly, the number of SGs was also greater in A315-mutant TDP-43 cells but not by a significant extent [Figs. 5(b) and 5(c)]. Excess Met treatment reduced SG volume in A315T-mutant TDP-43 cells dramatically but insignificantly in Wt cells. The numbers of SGs were also increased in both types of cells subjected to excess Met, but the changes were not significant [Figs. 5(b) and 5(c)]. On the other hand, both the volume and number of LDs were evidently larger in A315T-mutant TDP-43 cells than in Wt and control cells. Excess Met treatment dramatically elevated the volume and number of LDs in A315T-mutant TDP-43 cells and Wt cells compared with control cells [Figs. 5(d) and 5(e)]. Notably, under excess Met treatment, as LDs increased in volume and number, SGs decreased in volume yet increased in number, suggesting a potential interplay between the metabolism of these two subcellular organelles.

## 4 Discussion

Our study for the first time explored the antioxidant effects of a methionine-enriched diet on cells expressing TDP-43 using a multimodal optical imaging platform of DO-SRS and 2PEF microscopies. Specifically, using DO-SRS imaging, we revealed the cellular metabolic dynamics of lipids, proteins, saturated lipids, unsaturated lipids, and Cyt c. Using 2PEF imaging, we quantified the optical redox ratio through NADH and FAD. Furthermore, the volumetric SRS imaging of SGs and LDs demonstrated how single cells modulated the morphology of these organelles in response to stress. This work further extends our previous results by providing additional analyses of Cyt c, SGs, and LDs.^15^ In comparison with conventional metabolic imaging methods such as MS-based imaging techniques, our multimodal imaging platform overcomes the limitations in spatial resolution, enabling subcellular and quantitative imaging across various processes to comprehensively characterize the antioxidant effects of a methionine-enriched diet on cells expressing TDP-43.

Leveraging this cutting-edge imaging platform, we found that the A315T-mutant TDP-43 expression reduced global protein synthesis (protein turnover rate) under normal Met condition, aligning with previous studies that identify disrupted proteostasis as an early pathological hallmark of ALS.^20^^,^^32^ Although L-methionine and its metabolite SAM can enhance protein synthesis by promoting ribosome biogenesis and increasing mRNA translation into proteins,^33^ our study observed that excess methionine exacerbated the reduction of protein synthesis in TDP-43 expressing cells. This reduction of protein turnover rate was more pronounced in Wt than in A315T-mutant cells. However, protein synthesis in A315T-mutant cells remained significantly lower than in controls under all conditions, likely linked to the inherent impact of the A315T mutation in TDP-43 on cellular pathways such as disrupted proteostasis.^20^^,^^32^ Interestingly, excess levels of L-methionine can activate stress pathways and cause proteotoxicity, further lowering protein synthesis instead of rescuing it^34^—a phenomenon potentially relevant to ALS. In contrast, DO-SRS images showed that Wt and A315T-mutant TDP-43 expression increased *de novo* lipogenesis (lipid turnover rate) predominantly in the cytoplasmic region, where fragmented membranes induced by TDP-43 translocation are prevalent.^4^^,^^35^ Excess Met treatment further stimulated *de novo* lipogenesis in all cell lines to support cellular growth and proliferation. Notably, cells may deliberately reduce protein synthesis as a protective measure to conserve resources necessary for maintaining growth and mitigating damage through *de novo* lipogenesis under excess Met treatment. Future assays need to be conducted to investigate the biologically relevant molecules that govern these metabolic changes in cells expressing TDP-43 under the methionine-enriched diet.

Beyond the metabolic alterations in protein and lipid turnover rates, our multimodal imaging platform also uncovered how excess methionine could mitigate oxidative stress and its negative effects in TDP-43-expressing cells. NADH, an intrinsically fluorescent molecule, can be oxidized into non-fluorescent NAD+ in an elevated ROS environment, making the optical redox ratio (NADH/FAD) an indicator of oxidative stress.^23^ Our label-free 2PEF images demonstrated that TDP-43 expressing cells had significantly lower optical redox ratios than control cells, potentially resulting from disrupted mitochondrial complex activity, increased ROS accumulation, and reduced NADH levels caused by the translocation of aggregated TDP-43 from the nucleus to the mitochondria.^4^^,^^5^ Excess Met treatment increased the cytoplasmic localization of NADH, leading to a higher optical redox ratio in all cells. This effect was likely mediated by Met, which increased antioxidant defense through its metabolic conversion to glutathione and taurine, leading to ROS scavenging and NADH level restoration.

In response to oxidative stress, cells favor the conversion of unsaturated to saturated lipids because the latter contains single bonds that are less reactive to ROS.^36^ Our label-free SRS images showcased that TDP-43 expression encouraged this protective conversion under normal Met conditions. However, under excess Met treatment, unsaturated lipid levels were restored, corroborating previous studies that excess Met treatment effectively neutralized ROS through its direct antioxidant effects and metabolic conversion into glutathione and taurine.^6^^–^^8^ Because of this, the fatty acid imbalance was corrected after excess Met treatment. Single-cell analysis showed that the cytoplasm near the nuclear, where mitochondria are concentrated, exhibited higher lipid unsaturation compared with cellular membranes under both regular and excess Met treatments. Specifically, in regions with elevated ROS from dysfunctional mitochondria caused by TDP-43 expression, cells reduced unsaturated lipids to mitigate oxidative stress. Excess Met treatment reversed this effect, ultimately restoring mitochondrial structure and respiratory function through the production of unsaturated lipids. Future research should incorporate additional biochemical validation of oxidative stress and cellular repair. For example, the 4-HNE assay can be used to quantify 4-HNE levels, a stable byproduct of lipid peroxidation that accumulates under oxidative stress. Elevated 4-HNE levels indicate ongoing oxidative stress, whereas a reduction suggests effective cellular repair. In addition, identifying the specific subtypes of unsaturated lipids that increase following excess Met treatment could provide a deeper understanding of their role in reducing oxidative stress, restoring mitochondrial function, and potentially slowing ALS progression.

Accumulated ROS during oxidative stress can oxidize the heme iron in Cyt c from ferrous ($Fe^{2+}$) to ferric ($Fe^{3+}$),^37^ triggering its release from mitochondria and inducing apoptosis.^5^^,^^25^ Our label-free SRS images showed that Cyt c levels were the highest in A315T-mutant TDP-43 cells under normal Met conditions. This supports previous findings that A315T-mutant TDP-43 promoted ROS accumulation that could readily oxidize Cyt c.^4^ The increased Cyt c oxidation indicated elevated cellular apoptosis, which stemmed from mitochondrial dysfunction such as disrupted mitochondrial complex activity and altered redox state.^4^^,^^5^^,^^25^ Excess Met treatment likely enhanced antioxidant defense, thereby lowering ROS levels and reducing the likelihood of free radicals oxidizing Cyt c in A315T-mutant TDP-43 cells. The resulting decrease in oxidized Cyt c suggested that mitochondrial dysfunction and cellular apoptosis might be mitigated in diseased cells under excess Met treatment.

Expanding on our findings related to protein and lipid metabolism, we investigated how these metabolic changes recruit membraneless organelles such as SGs, which sequester misfolded proteins during stress,^26^ and LDs, which store lipids for energy,^27^ to combat cellular stress in TDP-43 expressing cells with volumetric SRS imaging. Under normal Met conditions, the expression of TDP-43 particularly induced the formation of SGs in cells. Such formation was not observed in the control HEK cells under the same condition. In accordance with prior findings, this evidence indicated that the expression of TDP-43 increased oxidative stress, which in turn prompted the formation of SGs.^38^^,^^39^ These membraneless organelles may act as a protective mechanism by sequestering damaged biomolecules, such as misfolded TDP-43 linked to ALS and ROS-oxidized protein, to prevent further cellular damage. This effect was more pronounced in cells expressing A315T-mutant TDP-43 compared with those with Wt TDP-43 as evidenced by a significant increase in SG volume under normal Met conditions, whereas the change in SG number remained relatively modest. Under excess Met treatment, the number of SGs increased (though insignificantly), whereas their volume decreased significantly between Wt and A315T-mutant TDP-43. A larger sample size is needed to assess the significance of the SG number. Nonetheless, this was evident in fewer damaged biomolecules produced under TDP-43 expression due to Met treatment, requiring smaller SGs for sequestration. Visualization of LD metabolism by SRS provided additional information on how single cells utilized this organelle to alleviate stress. Under normal Met conditions, Wt and A315T-mutant TDP-43 cells showed a significant increase in LD volume and number. Excess Met treatment further amplified LD expansion, which is believed to support *de novo* lipogenesis needed to restore growth and proliferation in these cells. It is crucial to note that as LDs increased in volume and number under excess Met treatment, SGs decreased in volume yet increased in number (even though insignificantly). This evidence offered direct visualization of how the formation of LDs may impact key proteins and signaling pathways, such as the mTOR signaling pathway, which are crucial for SG biogenesis.^40^ By altering these pathways through its increased metabolism under excess Met treatment, LDs may alter SG metabolism. Overall, both organelles can serve as metrics to measure single cells’ responses to TDP-43 expression as well as a methionine-enriched diet. Analyses of the underlying mechanisms dictating these morphological changes should be conducted to provide a better understanding of single cells’ response to TDP-43 expression and Met treatment.

Although our findings provide valuable insights into Met as a potential antioxidant in TDP-43-expressing cells, there are several limitations in the current study.

First, our study utilized fixed cells, which may have influenced the optical redox ratio due to protein cross-linking effects caused by 4% PFA fixation.^41^^,^^42^ This could potentially alter the fluorescence signals of NADH and FAD. However, as our study focused on relative redox differences between TDP-43-expressing and control cells, the observed variations remain valid. Future studies may incorporate live-cell imaging to track dynamic metabolic changes in response to TDP-43 expression and excess Met treatment, therefore eliminating potential fluorescence interference caused by PFA fixation.

Second, our study lacked additional validation of Met’s effects in *in vivo* models as *in vitro* models alone cannot fully capture the systemic effects of excess Met within a living organism. Future studies will transition from *in vitro* model to an ALS animal (*Drosophila*) model by introducing Met supplementation to assess its effects in a more complex biological system. The transgenic fly model will express the same wild-type (WT) and A315T-mutant TDP-43 as those used in the vitro TDP-43 in this study.

Third, our study did not compare Met’s antioxidant effects with those of established antioxidants and metabolic regulators in ALS. For instance, SAM, a derivative of L-methionine, has been shown to activate the antioxidant enzyme heme oxygenase-1 (HO-1), reducing lipid peroxidation and enhancing cellular defense against oxidative stress.^43^ In addition, a previous study suggests that a combination of vitamin E, selenium, and methionine can increase glutathione peroxidase activity and mitigate oxidative stress.^44^ Consistent with these studies, our findings indicated that excess Met promotes unsaturated lipid synthesis and NADH levels—both markers of reduced lipid peroxidation and oxidative stress. Moving forward, we aim to compare Met’s effects against known ALS antioxidants, such as SAM, vitamin E, and selenium, and explore the potential synergistic effects of combining methionine with these antioxidants as a strategy to slow ALS progression.

## 5 Conclusion

In summary, this study shows that L-methionine provides an antioxidant defense mechanism to TDP-43 expressing cells. At the same time, the metabolism of lipid synthesis and protein synthesis were also altered to promote cellular growth and repair in TDP-43-expressing cells under excess Met treatment. In addition, the treatment facilitated the conversion of saturated lipids into unsaturated lipids, addressing fatty acid imbalances. Cyt c oxidation was also reduced under the excess Met treatment, which consequently alleviated mitochondrial dysfunction and apoptosis in TDP-43-expressing cells. Furthermore, the treatment relieved stress responses in TDP-43 expressing cells by inhibiting SG formation through the enhancement of LD metabolism. Therefore, excess Met treatment could be employed as a potential supplement to slow down the progression of ALS. The non-invasive, multimodal DO-SRS and 2PEF imaging platform empowers us to visualize the spatial distributions of different molecules at a subcellular scale for a better understanding of ALS and the potential of methionine in treating ALS.

## Supplementary Material

10.1117/1.JBO.30.S2. S23906.s01

## Data Availability

The data generated in this study are available upon reasonable request from the corresponding author.
