# Supplementary material for: Optical imaging of metabolic dynamics in ALS under methionine regulation
Source: J Biomed Opt. 2025 May 24;30(Suppl 2):S23906. doi: 10.1117/1.JBO.30.S2.S23906 (PMC12102500; doi:10.1117/1.JBO.30.S2.S23906)

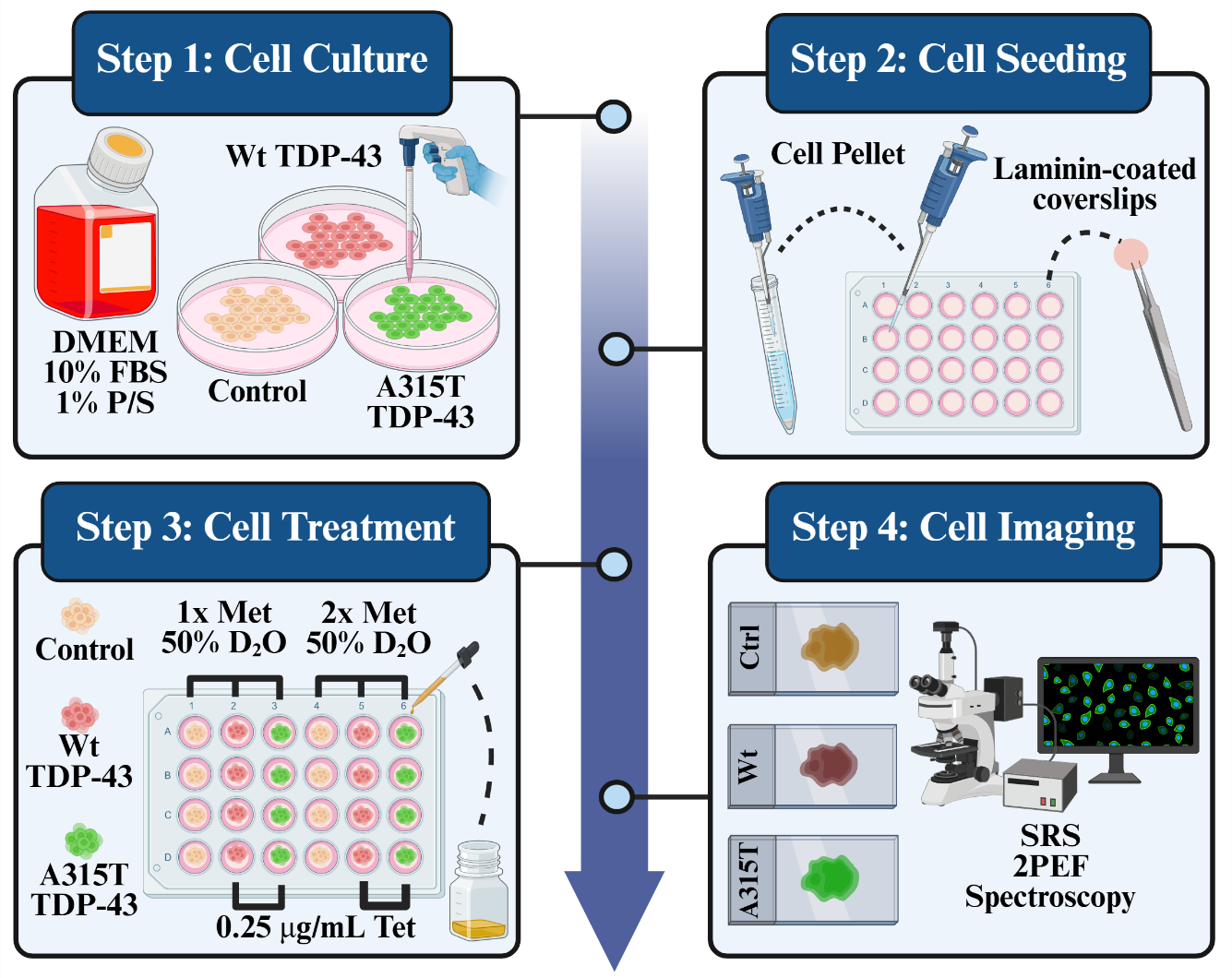


**Fig. S1** Cell culture configuration. In step 1, Control (Ctrl), Wild-type (Wt) or an ALS-associated TDP-43 mutant (A315T) cellular samples were cultured in DMEM medium containing 10% FBS and 1% P/S. In step 2, cells were seeded on laminin-coated coverslips for 12 hours before being synchronized with 0.5% FBS DMEM for 6 hours. In step 3, Wt and A315T-mutant TDP-43 cells were incubated with 0.25 μg/mL Tetracycline (Tet) in the DMEM medium for 36 hours to induce TDP-43 expression. All cells were then incubated in DMEM containing 50% (v/v) D2O with either 1x Methionine (1x Met) or 2x Methionine (2x Met) for 48 hours. After that, the coverslips were mounted on to microscope slides. In step 4, the cells were subjected to imaging with the Stimulated Raman Scattering (SRS) microscopy, Two-Photon Excitation Fluorescence (2PEF) microcopy, and Spontaneous Raman Spectroscopy


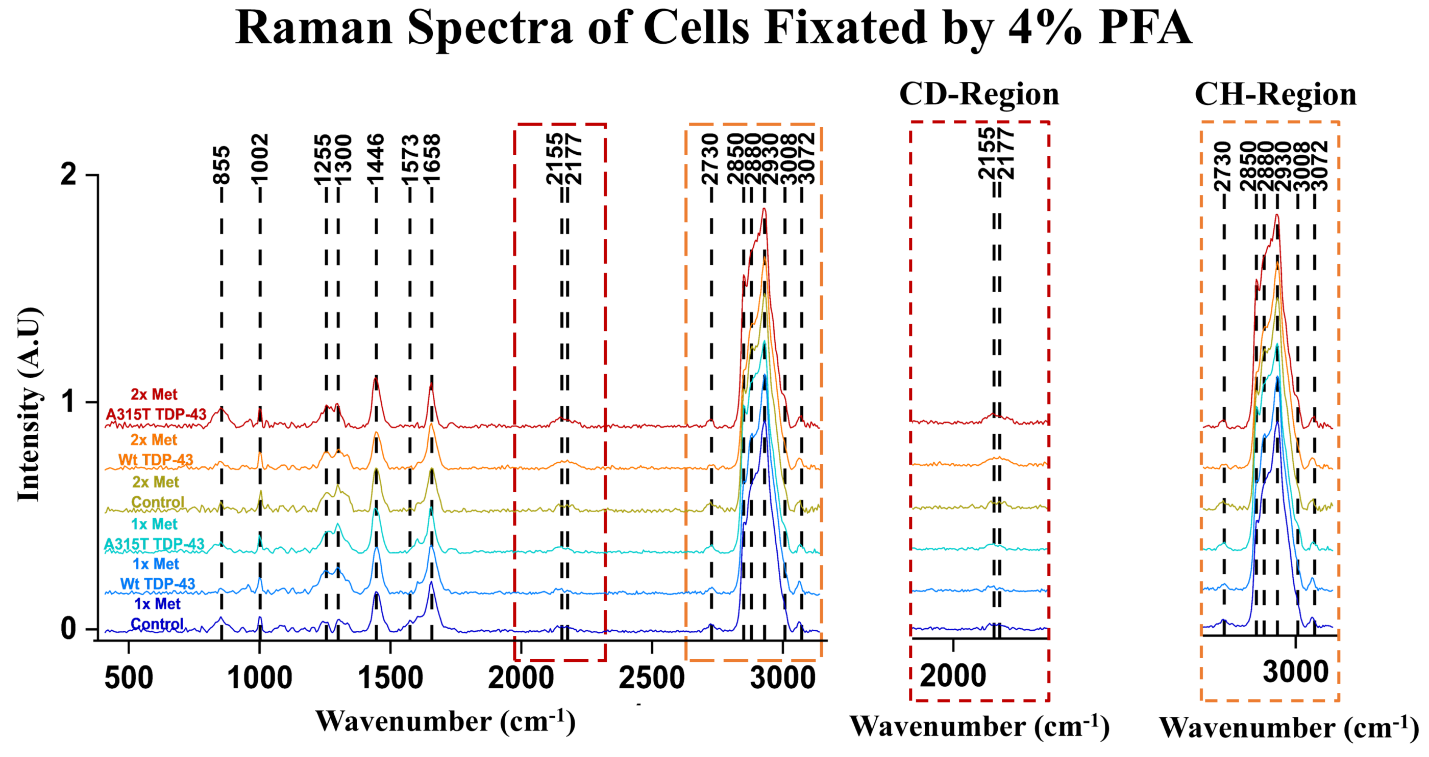


**Fig. S2** Raman Spectra of 4% Paraformaldehyde-fixated (PFA) cells. Spontaneous Raman spectra of cells fixated by 4% PFA fixation. The cells retain their metabolic profiles such as lipid and protein under 4% PFA fixation. Noticeably, all cells cultured in 50% D2O medium for 48 hours under regular (1x, 30 mg/L) methionine or excess (2x, 60 mg/L) methionine displayed C-D signals of newly synthesized lipids and proteins at 2155 cm-1 and 2177 cm-1, respectively.


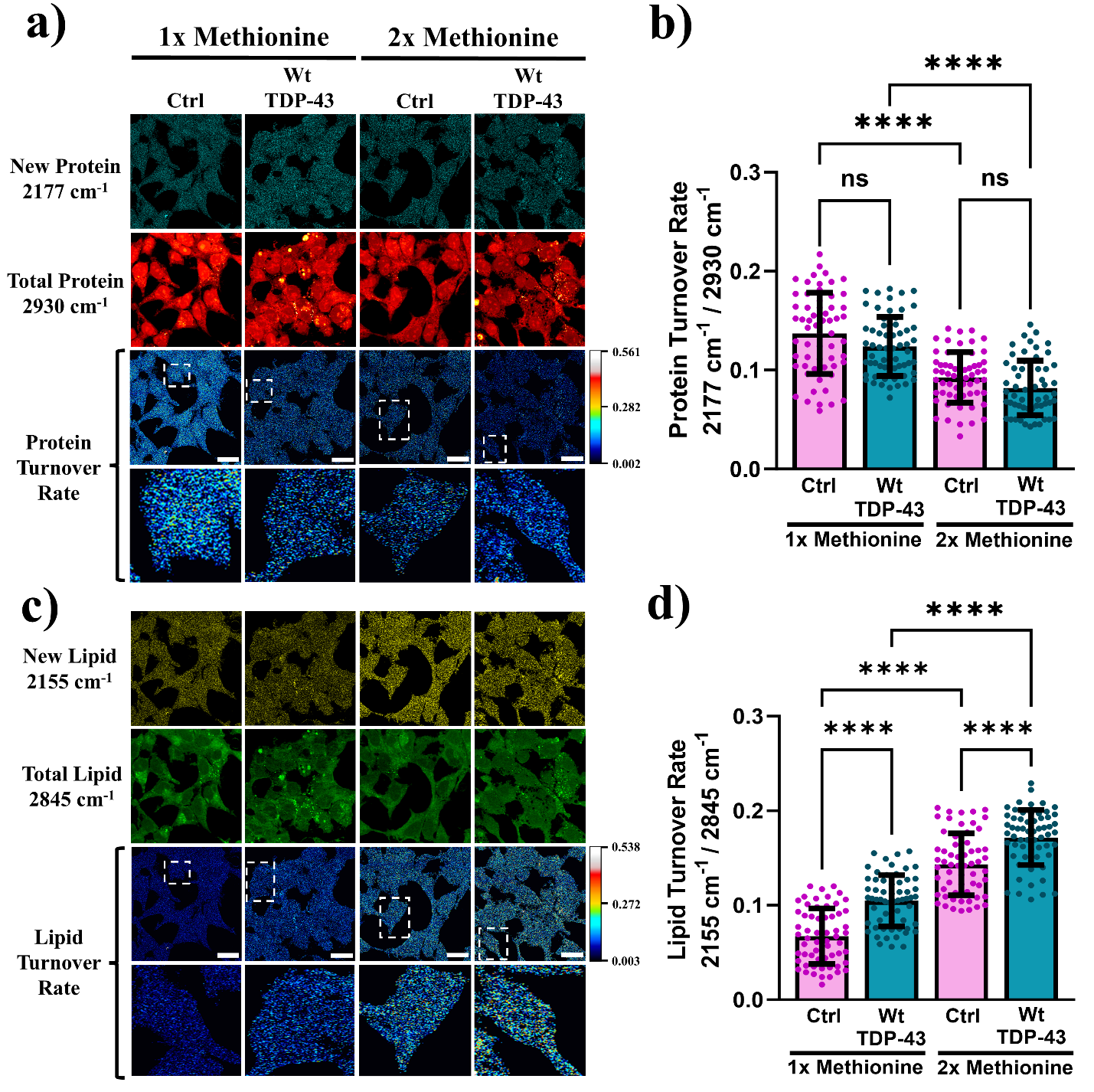


**Fig. S3** DO-SRS Imaging of Deuterated Lipids and Proteins in Control (Ctrl) and Wild-Type (Wt) TDP-43 Cellular Samples under Methionine Regulation. a) and c) DO-SRS images of newly synthesized protein and lipid in the Ctrl and Wt TDP-43 samples at 2177 cm^-1^ and 2155 cm^-1^, respectively. Cell samples were cultured in regular (1x, 30 mg/L) methionine or excess (2x, 60 mg/L) methionine medium, both supplemented with 50% D_2_O for 48 hours. b) and d) Quantitative analysis of the turnover rates for proteins (b) and lipids (d) in the Ctrl and Wt TDP-43 samples. Similarly, the turnover rates were calculated by dividing the amount of newly synthesized macromolecules by the total amount of existing and newly synthesized macromolecules. Data are presented as mean ± SD, with N = 60 cells per group. *, p < 0.05; **, p < 0.01; ***, p < 0.001; ****, p < 0.0001; ns, non-significant difference. Scale bar is 20 µm.


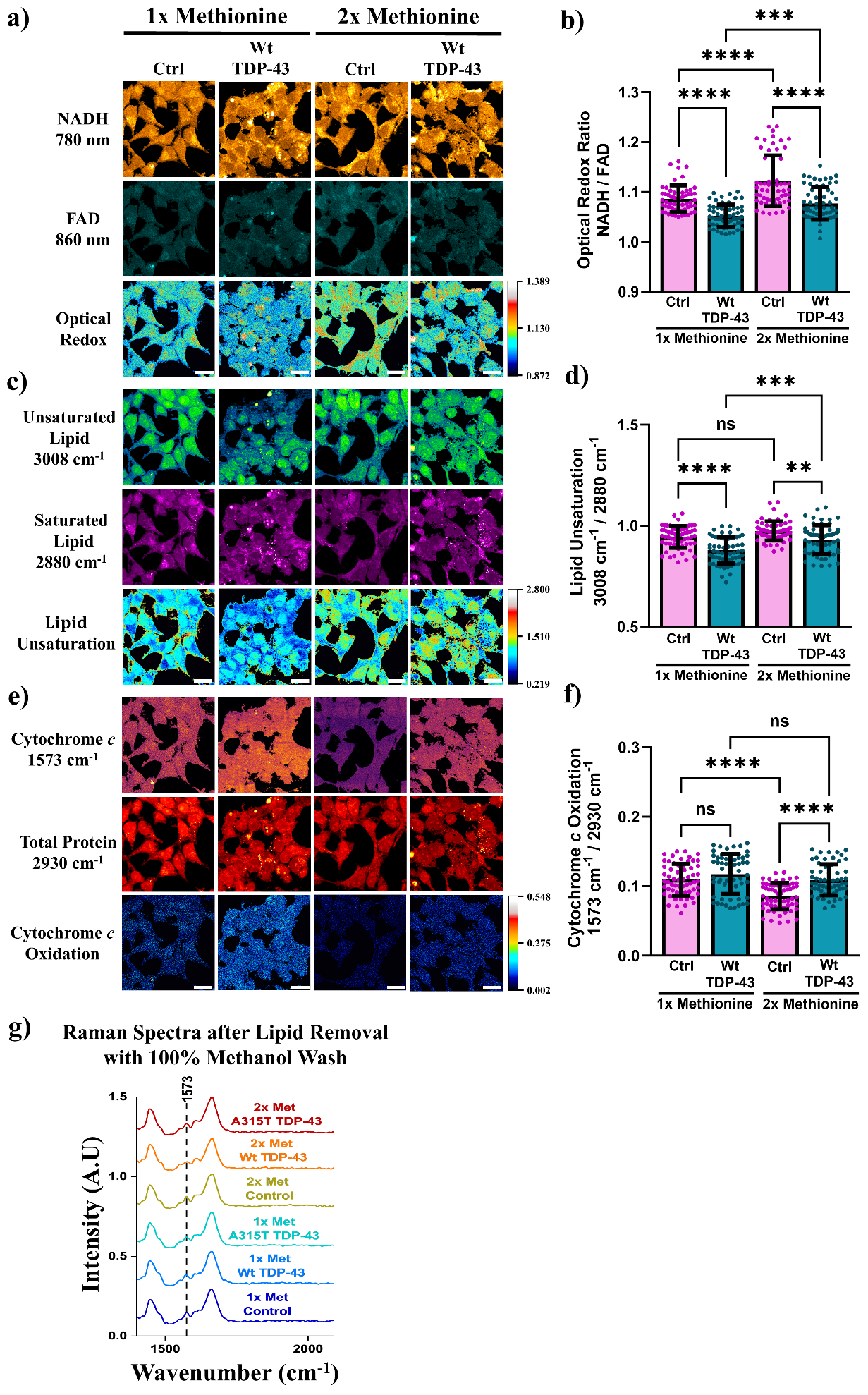


**Fig. S4** 2PEF and SRS Imaging of Cellular Redox State, Lipid Unsaturation and Cytochrome C in Control (Ctrl) and Wild-Type (Wt) TDP-43 under Methionine Regulation. a), c) and e) Label-free multichannel 2PEF and SRS images of the Ctrl and Wt TDP-43 samples under regular (1x, 30 mg/L) methionine or excess (2x, 60 mg/L) methionine medium. b), d) and f) Quantitative analysis of the optical redox ratio (b), lipid unsaturation (d) and cytochrome *c* oxidation (f) in the cell samples. With 2PEF, the optical redox ratio was determined by dividing the autofluorescence signal of NADH at 780 nm by the autofluorescence signal of Flavin at 860 nm. With SRS, lipid unsaturation ratio was obtained by dividing the amount of unsaturated lipid detected at 3011 cm^-1^ by the amount of saturated lipid detected at 2880 cm^-1^, meanwhile cytochrome *c* oxidation was evaluated by dividing the amount of oxidized cytochrome *c*  detected at 1573 cm^-1^ by the total amount of protein detected at 2930 cm^-1^ . g) Spontaneous Raman spectra of cells washed with 100% MeOH. The MeOH removed all lipid content in cells. The peak at 1573 cm^-1^ remained after MeOH wash belonged to a protein-rich molecule such as Cyt *c*. Data are presented as mean ± SD, with N = 60 cells per group for optical redox, lipid saturation and cytochrome *c* under different methionine conditions. *, p < 0.05; **, p < 0.01; ***, p < 0.001; ****, p < 0.0001; ns, non-significant difference. Scale bar is 20 µm.


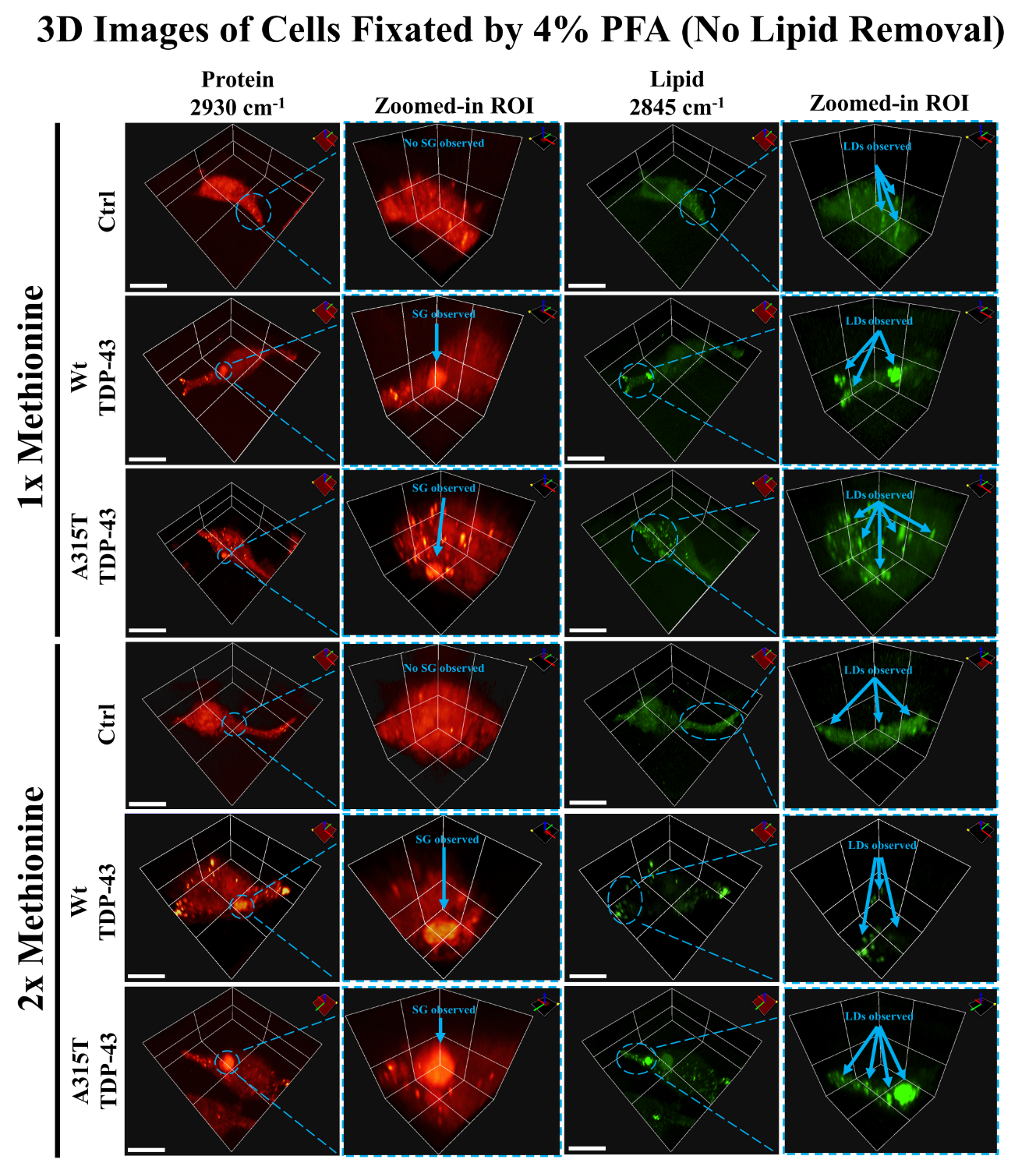


**Fig. S5** Visualization of Subcellular Organelles – Stress Granules and Lipid Droplets in 4% Paraformaldehyde Fixated Control (Ctrl), Wild-Type (Wt) TDP-43 and an ALS-associated TDP-43 Mutant (A315T) Cellular Samples under Methionine Regulation. Cells cultured in regular (1x, 30 mg/L) methionine or excess (2x, 60 mg/L) methionine medium retain their metabolic profiles of lipid and protein under 4% PFA fixation. Dashed outlines demonstrate single stress granules or cluster of lipid droplets that were observed on the same single cells at 2930 cm^-1^ and 2845 cm^-1^, respectively.

**Fig. S6** Visualization of Subcellular Organelles – Stress Granules and Lipid Droplets in Control (Ctrl), Wild-Type (Wt) TDP-43 and an ALS-associated TDP-43 Mutant (A315T) Cellular Samples under Methionine Regulation with 100% Methanol (MeOH) wash. MeOH wash dissolve all lipid-based molecules in cells cultured in regular (1x, 30 mg/L) methionine or excess (2x, 60 mg/L) methionine medium, leaving behind protein-based molecules. Dashed outlines demonstrate only single stress granules that were observed on single cells at 2930 cm^-1^. 3-D images acquired at 2845 cm^-1^ revealed traces of stress granules, but not lipid droplets.
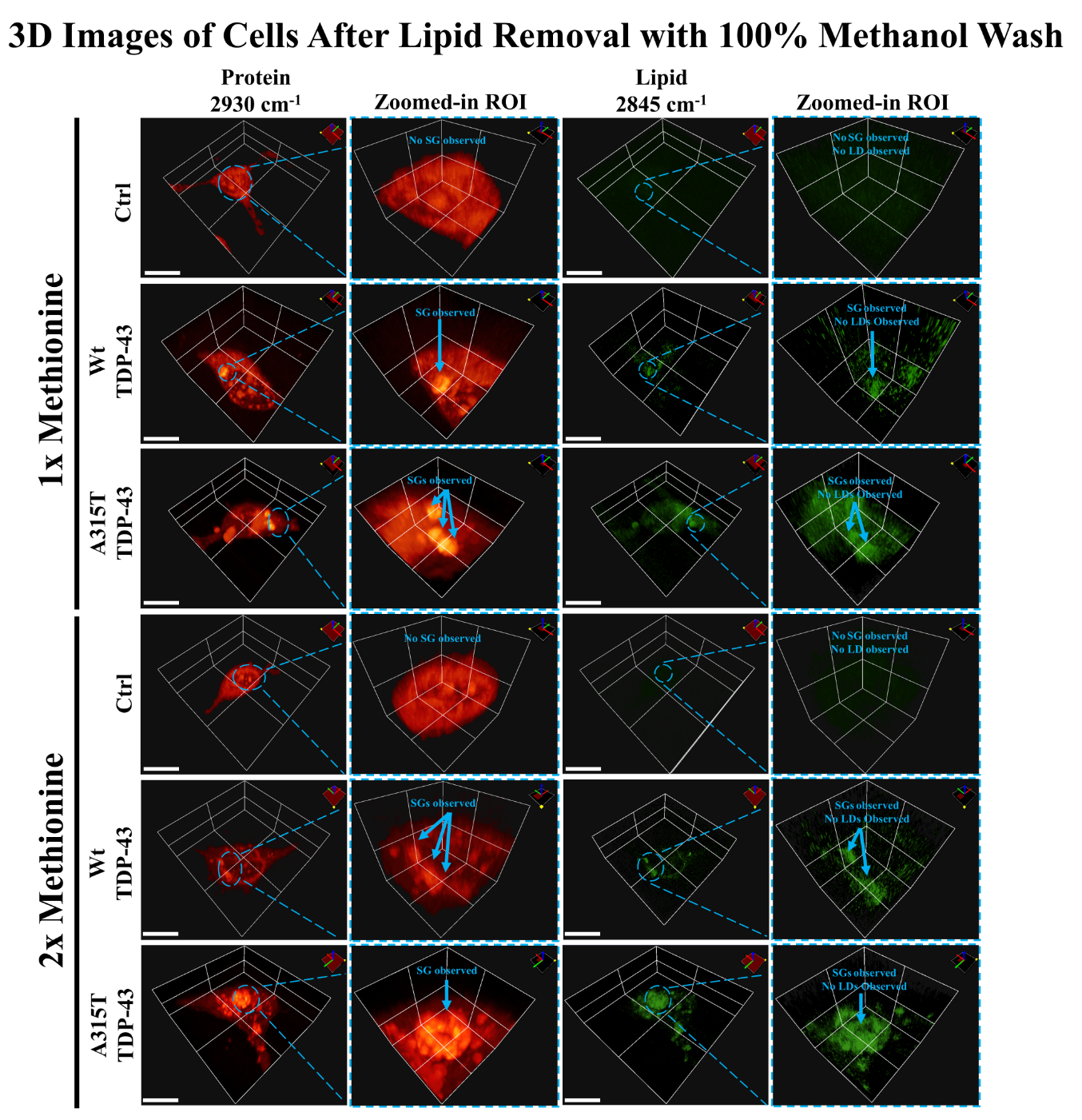

Supplement: Supplementary file 1 [file JBO_030_S23906_SD001.docx]
